# Stem cells and regenerative medicine in sport science

**DOI:** 10.1042/ETLS20210014

**Published:** 2021-08-27

**Authors:** Claire E. Stewart

**Affiliations:** Research Institute of Sport and Exercise Sciences, Liverpool John Moore's University, Byrom Street Campus, Liverpool L3 3AF, England, U.K.

**Keywords:** extracellular vesicles, injury repair, regenerative medicine, sport science, stem cells

## Abstract

The estimated cost of acute injuries in college-level sport in the USA is ∼1.5 billion dollars per year, without taking into account the cost of follow up rehabilitation. In addition to this huge financial burden, without appropriate diagnosis and relevant interventions, sport injuries may be career-ending for some athletes. With a growing number of females participating in contact based and pivoting sports, middle aged individuals returning to sport and natural injuries of ageing all increasing, such costs and negative implications for quality of life will expand. For those injuries, which cannot be predicted and prevented, there is a real need, to optimise repair, recovery and function, post-injury in the sporting and clinical worlds. The 21^st^ century has seen a rapid growth in the arena of regenerative medicine for sporting injuries, in a bid to progress recovery and to facilitate return to sport. Such interventions harness knowledge relating to stem cells as a potential for injury repair. While the field is rapidly growing, consideration beyond the stem cells, to the factors they secrete, should be considered in the development of effective, affordable treatments.

## Introduction to stem cells

To interrogate the potential of stem cells in sport science, we first need to gain oversight of the stem cells, themselves. In 1838, Matthias Schleiden and Theodor Schwann reported that the cell is the central unit of a biological organism and that ‘organisms only grow by the growth and division of single cells' (reviewed in [1]). Twenty years later, Rudolf Virchow extended the cell theory that all cells arise from another cell suggesting that ‘every animal appears as the sum of vital units, each of which bears in itself the complete characteristics of life’ (detailed in [2]). While many other researchers were providing critical evidence to this developing knowledge, perhaps what was less apparent, was that these researchers were already defining some key characteristics of stem cells — self renewal and specialisation.

The subsequent growth of the field has been vast, with claims that stem cells are capable of curing everything from hair loss to brain damage. A google search for: stem cell treatment returned 436 000 000 hits in less than a second. Turning to peer-reviewed articles, Google Scholar returned 3 110 000 hits and clinicaltrials.gov reported 8465 trials using stem cells. This therefore begs the question, what are stem cells and what is their potential?

**Figure 1.**
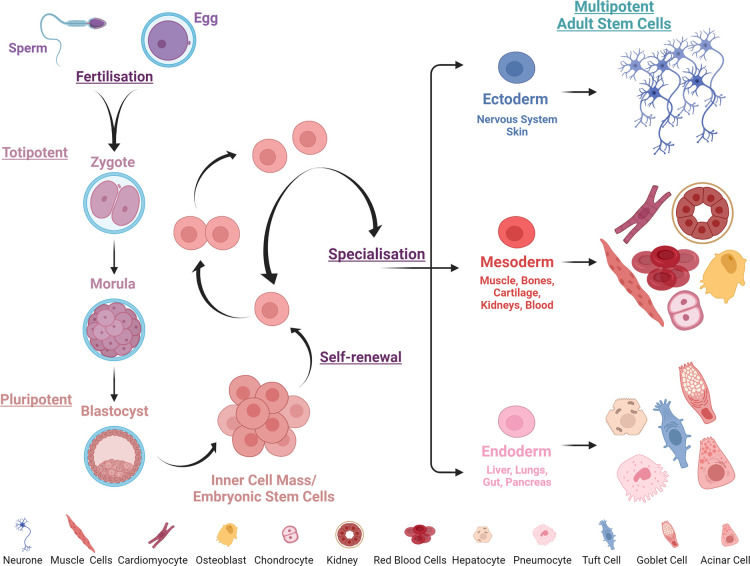
Illustration of the potential and the potency of embryonic and adult stem cells. Created with BioRender.com.

Both embryonic and adult stem cells exist *in vivo*. Embryonic stem cells are characterised by an ability to divide indefinitely and to differentiate (develop) into all cells within the human body. This ability is referred to as potency and is used to define the capacity of stem cells (Figure 1). Totipotent stem cells are derived from the fertilised egg, are the least specialised stem cells and can form all cells of the developing organism. Like totipotent stem cells, pluripotent stem cells (first isolated from mice in 1981 [3,4] and from humans almost 20 years later [5]) can form all tissues of the body, except the placenta — they are often referred to as embryonic stem cells (ESCs) and are derived from the inner cell mass (∼30–50 cells) of the pre-implantation embryo. Multipotent stem cells, found within adult tissues (identified in skeletal muscle [6] and bone marrow [7] in 1961), are more specialised and are lineage restricted, differentiating into cell types linked to their tissue of origin [8], with roles to facilitate maintenance and repair of adult tissues (Figure 1).

Different cells express different factors (e.g. proteins, miRNA, metabolites) and their phenotype (appearance) and behaviour are established by the factors which they express and the factors which they respond to. The potential of such knowledge was first reported in the mid-1980s, in seminal work of Harold Weintraub, who determined that myoblast determination protein 1 (myoD), expressed only in skeletal muscle, is a master regulator of myogenesis (muscle formation) [9–11]. Such research, in my view, enabled the monumental discovery by Takahashi and Yamanaka in the mid-2000s [12] that the forced expression of four key embryonic-like proteins (octamer-binding transcription factor 4 (OCT4), sex determining region Y-box 2 (SOX2), Myc, and Kruppel-like factor 4 (KLF4)), in adult murine (mouse) fibroblasts, could transform adult cells to an induced (embryonic-like) pluripotent stem-cell (iPSC) fate, with the capability to generate cells of all three germ layers. One year later, similar results were achieved using human fibroblasts [13,14]. The potential of these cells in advancing understanding in the arenas of developmental biology, medicine and big pharma attracts a lot of attention and the field is growing rapidly (see the following for some of the most recent examples for reviews on iPSCs and neurones [15,16], cardiomyopathies [17], neuromuscular diseases [18], diabetes [19], cardiac reconstruction [20], disease modelling [21] and even human evolution [22]. These are by no means intended to be exclusive or exhaustive references, merely current examples of potential). It is beyond the scope of this mini review to focus on ESCs or IPSCs in sport, instead the potential of adult stem cells will be explored.

## Exercise and stem cells

How our bodies respond to acute and chronic bouts of exercise loading (intensity, mode, frequency, duration), determines training programmes to enhance athletic performance and to facilitate recovery. In a sporting context, peak performance is the goal. Focus on age, gender and the impact of mechanical, thermal and nutritional loads on the musculoskeletal, pulmonary, cardiovascular, nervous, endocrine and metabolic systems are often investigated. Frequently, it is the physiological response to exercise interventions that are determined, however, the mechanisms underpinning adaptations which improve performance and facilitate recovery are key and occur at a cellular level. Therefore, to optimise performance and to facilitate recovery/repair, we need to understand which and how adult stem cells respond to exercise [23] and what role they may play in repair and regeneration following injury.

### Exercise and cellular communication

Multicellular organisms require elaborate networks of communication to co-ordinate their behaviour. Such co-ordination is required for function and survival. Higher organisms like ourselves are like ‘cellular cities', where groups of cells (e.g. neurones (ectoderm), myoblast (mesoderm), hepatocytes (endoderm)) perform specialised functions, which are linked by intricate communication systems. In addition to being the building blocks of our bodies, cells respond to internal/external stimuli and subsequently provide the communication enabling adaptation, function, repair and survival. Modes of communication may be local (autocrine or paracrine) or more distant (synaptic or endocrine). How does this relate to sport? The ability to move, let alone perform, requires a high level of co-ordination. Although muscle contraction allows for bone movement and hence locomotion, motoneurons (ectodermal) are needed to enable muscle (mesodermal) contraction, the endocrine system to facilitate neuronal activation and transduction as well as e.g. nutrient sensing, the cardiovascular (mesodermal) and hepatic (endodermal) systems to enable oxygen and nutrient delivery/waste removal. Cross talk between tissues is key and will drive adaptation at a cellular level. For example, secretion of myokines, by skeletal muscle in response to exercise [24,25], will result in: 1. increased insulin sensitivity, beta-oxidation and glucose uptake in the liver, 2. insulin sensitivity and beta oxidation in white adipose tissue, 3. insulin sensitivity, glucose uptake and hypertrophy (increased mass as a result of increased cross-sectional area) in skeletal muscle and 4. cardio-protection [26]. Such cross talk and adaptation are indicative of exercise influencing cellular behaviour in tissues derived from all three germ layers (ectoderm, mesoderm, endoderm). Furthermore, the fact that exercise is capable of inducing tissue remodelling suggests that adult stem cells are associated with exercise-induced adaptation [27]. What is the evidence?

### Impact of exercise on stem cell behaviour

**Figure 2.**
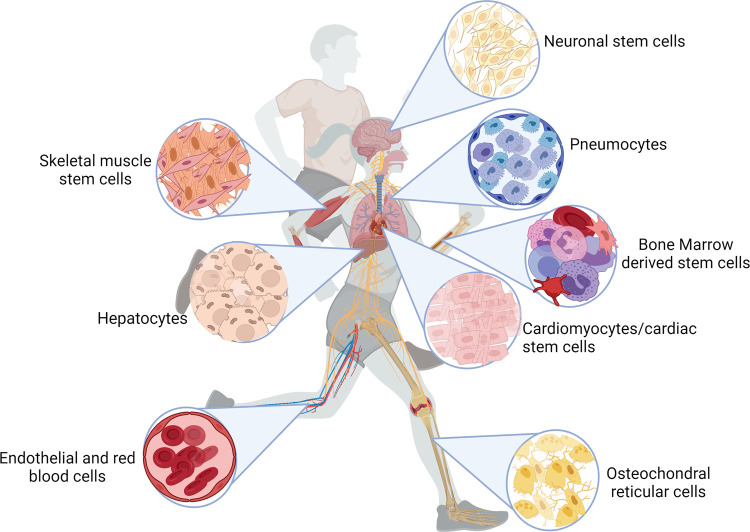
Illustration of the influence of exercise on adult stem cell expansion. Created with BioRender.com.

It is now well accepted that exercise provides a relevant stimulus to induce change (Figure 2), culminating in priming/activation of adult stem cells (for early review see [23]). Indeed, the potential positive impact of exercise on haematopoietic stem cell mobilisation was first reported by Barrett *et al*. in the late 1970s [28]. Subsequent studies suggested this process was age, intensity and time dependent (reviewed in [27]). Approximately 3 decades later, in the late 2000s an explosion of literature relating to exercise and stem cells appeared on the research landscape, with relevance to all systems involved in exercise adaptation.

For example, research by Kempermann et al. [29] in murine models, indicated that neuronal ageing, was reduced by sustained physical activity across the lifecourse, as a result of hippocampal neurogenesis. Further work in humans reviewed by Hillman et al. [30] indicated that moderate-intensity exercise benefitted brain function and cognition, not only in old age, but across the lifespan and also in the presence of neurodegenerative diseases [30]. This knowledge resulted in further studies by Blackmore et al. [31] who illustrated that moderate-intensity exercise in mice, significantly increased neural stem cell number and regeneration capacity in a growth hormone dependent manner.

If the brain is capable of responding to exercise in such a manner, then it is perhaps not too surprising that other tissues, or more specifically their resident adult stem cells, can too. Like the brain, traditionally, it was believed that the heart was incapable of repair, although endurance exercise was accepted to induce hypertrophy via growth of cardiomyocytes [32,33]. In 2010 Waring et al. challenged this concept, in relation to exercise adaptation. They reported that not only did intensity-regulated endurance running in rats culminate in *de novo* capillary formation, but also in cardiac remodelling. Critically, this was not only via hypertrophy of existing cardiomyocytes (as traditionally believed) but also via hyperplasia (increased cell numbers) of resident cardiac stem cells [34], thereby revealing the importance of exercise-dependent activation of resident cardiac stem cells in cardiac remodelling.

Such adaptations do not occur in isolation and indeed, research of Baker et al. in 4 week old juvenile mice, revealed that a 10 week progressive endurance training programme was capable not only of stimulating medullary haematopoiesis by up to 8 fold, but that it resulted in adaptation of the bone marrow niche, potentially as a consequence of cytokines expressed by and released form skeletal muscle [35] highlighting the importance of cross talk in adult stem cell behaviour.

It is well accepted that as we age, we lose muscle mass and strength (sarcopenia) and that such decrements impact negatively on function and quality of life. Exercise, particularly resistance training, can reduce the rate of decline [36]. It is believed that the impact of exercise on hormonal adaptation (e.g. Testosterone, Growth Hormone and the Insulin-like Growth Factors), in association with appropriate nutrition, results in stimulation of anabolic signalling pathways, which culminate in increased protein synthesis and hypertrophy [37]. The increased myonuclear domain, facilitated following e.g. eccentric lengthening contractions, is believed to be provided by activation, proliferation and myonuclear donation from resident muscle stem cells (satellite cells) [38,39]. Furthermore, data also exist to demonstrate that following eccentric exercise, pericytes accumulate in skeletal muscle and are believed to facilitate satellite cell expansion [40], another indicator of co-operation between the systemic and local environments to facilitate appropriate adult stem cell adaptation in response to exercise.

Since these discoveries relating to adult stem cell adaptation following exercise, the focus of the exercise world on stem cell behaviour has been on the identification of specific stem cell pools, their roles in adaptation and potential mechanisms of activation. Access to adult stem cells in humans is restricted by tissue accessibility therefore much research has focussed on skeletal muscle (biopsies) stem cells and circulating haematopoietic, endothelial and mesenchymal stem cells. While these are all impacted by mode and intensity of exercise, recent evidence (reviewed in [41]) suggests that future directions should focus on the timing and the extent of stem cell mobilisation, within and between the different stem cell sub-populations. To achieve this, it is suggested that agreement on cell surface markers, defining the sub-populations is key.

Mechanisms focussing on the epigenetic (lifecourse/lifestyle) influencers of stem cell dynamics basally [42] and in response to exercise [43] have generated extensive interest. Indeed the impact of training and detraining [44] and ageing [45] on epigenetic adaptation, as well as the influences of epigenetics on anabolism [46] in skeletal muscle is currently of fundamental interest [47]. Investigations into the roles of miRNAs in exercise-induced muscle adaptation [48] as well as exercise-induced exosome release and the role of the cargo on muscle adaptation [49] are all also gaining traction. Accessibility to extracellular vesicles in the circulation, together with an understanding of their site of generation may well lead the way in the coming decade in terms of knowledge growth, systems biology, exercise and adult stem cells — overcoming the issues of tissue accessibility. The potential of these and further omics-based approaches may well lead the field in terms of joining exercise physiology, stem cell biology and molecular mechanisms of change [50] as we enter the second quintile of the 21st century.

### Potential for stem cells for enhancing performance

While we are making huge progress in our understanding of the role that exercise plays in stem cell activation and in turn the roles that stem cells play in physiological/endocrine adaptation following exercise, the question often arises as to whether such knowledge could be applied to stem cell transplantation to enhance sporting performance. The immediate follow up questions should be: would it be ethical; how could it be regulated and would it be banned in sport by the world anti-doping agency (WADA)?

With that in mind, it is well known that endurance performance may be improved by increased red blood cell volume. This has culminated in blood doping — EPO administration, synthetic oxygen carriers, autologous (donor and recipient are the same person) blood transfusions — all of which are banned in sport, under WADA's list of prohibited substances and methods (https://www.wada-ama.org/en/questions-answers/blood-doping). In contrast, living or training at altitude (exposing the body to an hypoxic environment), then competing at sea level may provide a beneficial impact on performance, particularly via an increase in red blood cell volume, albeit impacted by: e.g. time at altitude, level of altitude, athletic performance at altitude and individual variability. If benefits are acquired, they tend to occur at multiple levels including: haematological, cardiovascular, ventilatory, increased capillary density, improved muscle function/metabolism and increased myoglobin abundance [51]. Therefore, while such training may confer an edge, mediated by adaptation at a cellular and tissue/organ level, the need to train hard still prevails.

The use of stem cells in the cardiovascular arena has been with a focus on post-myocardial infarction, ischaemic (oxygen deprivation) heart disease and cardiomyopathy (enlarged, rigid heart), rather than to elicit beneficial exercise-induced adaptations. Initial transplant studies by Menasché relied on the use of autologous skeletal muscle stem cells in the treatment of ischaemic cardiomyopathy [52,53]. These studies were closely followed by trials using autologous bone marrow derived stem cells for transplantation, including the REGENERATE trials for ischaemic heart disease (IHD) and dilated cardiomyopathy (DCM) [54,55]. While these were heralded as providing the potential for life changing interventions, despite subsequent studies being large scale, randomised, placebo controlled and double blinded, the beneficial impact of such early transplant trials were equivocal [56]. The lack of an overt benefit was believed to be as a result of heterogeneity (diversity) of the cell populations being transplanted, very poor capacity for engraftment and limited differentiation potential to cardiomyocytes [57]. Almost 15 years later, the field has progressed, with cardiac-committed and pluripotent stem cells being investigated [58], although homing, engraftment and cell type are still an issue. Fifteen years have elapsed since stem cell transplantation was conceived to be the cure for heart diseases, despite the extreme clinical need and the huge financial investment, the lack of a successful intervention, together with the high cost, make this an unlikely option to improve cardiovascular performance in the sporting world.

What about the potential for stem cell transplantation in power-based sports? Could stem cell transplantation be used to increase muscle mass and thereby strength? The average, healthy, young human will have ∼40–50% of their body mass as skeletal muscle. It functions to enable growth, posture, locomotion, thermoregulation, metabolism and survival. Muscle mass and strength can increase as a result of resistance exercise training. Exercise evokes adaptation at an endocrine level resulting in increased expression of anabolic hormones (decreased levels of catabolic hormones), by stimulation of hypertrophic and hyperplastic signalling pathways (e.g. mTOR, Akt, ERK), through increased satellite cell activation/division and increased net protein synthesis. The ultimate output is larger muscles and increased strength (for review see [59]). Although speculation abounds as to whether muscle stem cell transplantation could benefit hypertrophy in power-based sport, the sheer volume of muscle, in a healthy individual, would necessitate lengthy cell culture periods to produce a fraction of the stem cells needed for transplantation. These obstacles, together with issues of poor homing and engraftment as well as astronomical costs evoked, make stem cell transplantation, in power-based sport, an unlikely option. Hormone administration (in the absence of disease) to benefit performance is already banned by WADA. Does this mean there is no potential for stem cells in sport? What about the repair of injury?

### Stem cells and repair of injury

Soft tissue injuries can be catastrophic for the afflicted athlete, compromising performance and even ending careers. Furthermore, the costs can be vast; in the 2019/20 season, the average annual salary of the top 10 highest paid first team players in the English premier division was £4.5 million/year, with the average salary of a first team player at Manchester City (the highest paying club) being £6.99 million/year. Given that the average duration of absence following injury, in UEFA elite club football, is up to four weeks per injured player [60], then at a salary cost of ∼£135 000 per week, it is a huge financial burden to the club to have an injured player on the bench. In the absence of surgical intervention, rest and rehabilitation are the current options for recovery, with an associated risk of bringing players back before they are fully recovered, thereby increasing the threat of re-injury.

The potential for stem cells in the repair of injury is an alternative arena, gaining great traction. Regenerative medicine in the sporting arena focusses predominantly on cartilage, tendon, ligament, meniscal and skeletal muscle injuries. In addition to the potential of stem cells themselves in the repair of injury, platelet rich plasma (PRP), tissue engineering and extracellular vesicles are also investigated. These are explored further in the example tissue injury models below.

#### Injured tendons: potential of platelet rich plasma

Most tendons attach muscle to bone, transmitting mechanical forces from the muscle to the bone and enabling movement. They are composed predominantly of type 1 collagen fibres and proteoglycans (proteins with sugar residues attached), which give them their high tensile strength and viscoelasticity, respectively. Resident tenocytes (mature tenoblasts — immature, highly proliferative cells) are believed to produce growth factors (e.g. transforming growth factor beta (TGFβ1) and scleraxis (SCXA)) that stimulate collagen synthesis, as well as collagen itself, in response to load/strain [61]. Since adult human tendons are believed to contain multipotent stem cells [62], the potential for self-repair does exist; however, tendons have a limited healing process and are prone to scarring. The inadequate understanding of markers delineating tenocytes from tendon-derived stem cells, has slowed progress with regards to endogenous (local) stem cells and tendon repair. It is therefore the focus of extensive current research (summarised in: [63]).

All soft tissues (e.g. tendon, muscle, cartilage, ligaments), impacted significantly by injury in the sporting arena, can repair. However, this process is often slow and inadequate, with scarring of the injured tissue culminating in reduced function and often significant re-injury. The goal, therefore, would be to improve the repair process from a temporal (rate of repair) and functional perspective.

Ideally, the injury would not happen in the first place. Specific training/recovery regimes should be considered, for those players prone to injury, to ensure optimal and sustained performance. Never the less, tendon injuries constitute ∼40–50% of all sporting-related injuries, making this a fundamental issue warranting further investigation. Despite their capacity to regenerate, successful repair is poor, potentially due to their low vascularity. This would impact negatively on clearance of damaged tissue, immune infiltration and inflammation, systemic regulation of the damage/repair process and potentially activation of resident stem cells. Conventional interventions therefore rely on surgery, physiotherapy and anti-inflammatory drugs (which may compromise the repair process further) for pain management. Given the known difficulties of enabling exogenous (expanded outside the body for transplantation purposes) stem cell homing and engraftment (worsened in the absence of good blood supply), alternative options are being sought to enable the endogenous processes of repair. Since platelets play an important role in wound repair, PRP has been investigated for its potential in tendon injuries. Early studies suggested PRP was capable of facilitating tendon repair. This was believed to be because of the differentiation of tendon stem cells to tenocytes as a result of secondary release of growth factors in the injured area [64]. Given the autologous nature and ease of delivery of such treatments, there has been a real research focus on such interventions. Both PRP and leukocyte-enriched PRP interventions have been studied. The latter reportedly evoked improved pain relief, neovascularisation and function, compared with the former, however, timing of administration (leukocyte-enriched PRP administration can be detrimental if provided late, not early in the repair process) and mode of preparation are key [65]. Moving away from stem cell transplantation towards facilitated repair by local stem cells may be the way forward for tendon injuries in future. This should be taken with caution, however, as large-scale studies remain to be performed to confirm or refute the benefits of such treatments.

#### Cartilage injury and tissue engineering

**Figure 3.**
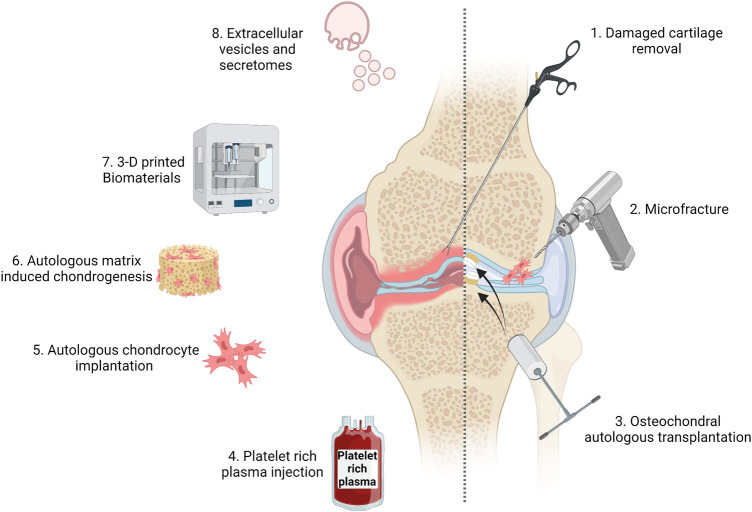
Illustration of the development of interventions for use in sporting injuries (e.g. cartilage damage) from stem cells to bioengineering. Created with BioRender.com.

The cartilage is mainly found between bones in joints. They absorb shock and enable joint mobility and durability via the provision of frictionless movement, which allows load transmission. Like tendons, cartilage is regularly damaged in sporting injuries. This compromises movement and induces significant pain. Furthermore, ligament strains and rupture frequently culminate in joint dislocation and significant cartilage damage. Cartilage is made up of slow growing cells called chondrocytes. These cells secrete collagen, proteoglycans and elastin, all of which provide cartilage with its functional properties. However, cartilage does not have a blood supply (compromising oxygen and nutrient delivery), which together with the slow growth rates of chondrocytes means its ability to repair is compromised. The high prevalence of cartilage injuries, together with its relatively inefficient repair capabilities mean that historically the most frequent interventions relied on damaged cartilage removal (Figure 3). This will allow short term relief, but is associated rapid degeneration of any remaining non-damaged tissue and ultimately early onset osteoarthritis. More recently, approaches to retain uninjured cartilage, while promoting regeneration or facilitating repair via transplantation prevail. Different methods are evolving depending on the size and scale of injury [66].

In patients with small cartilage defects, microfracture (Figure 3) is frequently employed. This relies on drilling small holes into the subchondral bone, which allows mesenchymal stem cells from the bone marrow to migrate to the injured site, where they lay down collagen and improve joint function. Unfortunately, these cells tend to secrete type I collagen, similar to fibrocartilage, not type II collagen, which is present in hyaline cartilage of most joints. Therefore the long-term success of such interventions, potentially as a result of the cartilage make-up, is more limited [67], particularly in the sporting world [68]. The use of microfracture has progressed to interventions using nano-fractures, although results in humans remain to be determined (for review see [66]).

Such technologies have further developed to include osteochondral autologous transplantation (Figure 3). Plugs of cartilage from non-weight bearing joints are transplanted to the site of injury, however, integration of the transplanted tissue and degeneration with time, cause sustained complications on a patient by patient basis. Therefore these interventions have progressed to include: 1. autologous chondrocyte implantation (ACI), 2. matrix-induced ACI (MACI), where autologous stem cells are expanded in culture, seeded into collagen and transplanted into the inured site and 3. autologous matrix-induced chondrogenesis (AMIC), which involves combining microfracture with cell free collagen transplantation at the site of injury [69].

A systematic review of randomised controlled trials (RCTs) of the most appropriate surgical treatments for knee cartilage defects, investigated data from 10 publications, including 861 patients. It reported that at 10 years post intervention, significantly more failures occurred with microfracture compared with osteochondral autologous transplantation and more occurred with osteochondral autologous transplantation compared with ACI. Larger lesions (>4.5 cm^2^) treated with cartilage regenerative techniques (ACI/MACI) had better outcomes than with microfracture. Based on the evidence of the review, authors concluded ‘no single treatment can be recommended for the treatment of knee cartilage defects. This highlights the need for further RCTs, preferably patient-blinded, using an appropriate reference treatment or a placebo procedure' [70]. Despite this conclusion, cartilage tissue engineering does hold promise, with a need to improve knowledge relating to key chondrogenic factors, the number of transplanted chondrogenic cells, the biocompatibility of the engineered matrices/scaffold materials being used and the size and type of injury. The inclusion of 3D printed biomaterials (Figure 3) to improve transplantation success is also being investigated [71].

#### Musculoskeletal injury and extracellular vesicles

The inclusion of stem cells, in injury repair models, together with improved delivery using biocompatible scaffolds is heralded as the way forward for many musculoskeletal injuries. However, it is known that cell homing and integration are problematic, as is the lifespan of the injected stem cells. Therefore, while this arena develops, others are investigating the potential of extracellular vesicles in the repair of injury. Extracellular vesicles are small cell membrane derived particles, transported throughout the body via the circulation and carrying cellular cargo from one site to another in the body (for detailed description see [72]). Some of the earliest data illustrating that cardiac myocytes release extracellular vesicles were generated in the mid-2000s [73], since then the direct potential of stem cell transplant in the heart has been questioned and a role for paracrine signalling between the transplanted cells and the recipient tissue, facilitating endogenous repair by means of e.g. extracellular vesicles, is being investigated.

Studies of the role of extracellular vesicles in tissue repair extend beyond the heart, with a role for them being reported in both skeletal muscle [74] and bone [75] homeostasis, development, injury and regeneration. More specifically, investigations using extracellular vesicles implanted in scaffolds for the treatment of calvarial defects have reported improvements associated with increased bone mineral density and volume [76]. The use of similar interventions to treat skeletal muscle injuries (direct intramuscular injection or intravenous injection) suggested muscle repair associated with increased expression of muscle-specific transcription factors, increased fibre cross-sectional areas and decreased scarring [77]. If such interventions do indeed prove to be efficacious, then the use of stem-cell based secretomes or recapitulated secretomes would significantly improve recovery from injury, not just in the sporting arena, but also more generally following musculoskeletal injuries.

## Summary

- The use of stem cells in regenerative sport medicine is a rapidly growing arena with vast potential, if successful. Key to conclusions being drawn is an understanding of need, a definition of success and large scale, double blinded, multi-centre clinical trials.
- For stem cell transplantation to be beneficial, the recipient must be considered in terms of age, gender, lifestyle, activity/inactivity and multi-morbidities. One size fits all approaches may not work.
- Assessment of post-intervention needs are fundamental to the classification of ‘success' of stem cell transplantation/regenerative medicine. For example, is the recipient an elite athlete already at the end of their sporting career wishing simply to maintain a healthy active life or someone just starting out as an elite athlete in whom long term function under extreme stress and strain is key.
- Consideration to the extent and site of injury must be given — is this a micro-injury in a tissue that is capable of good self-repair (e.g. eccentric damage in skeletal muscle) or is it a catastrophic injury in a tissue incapable of efficient repair (e.g. cartilage injury).
- In terms of future directions, it must be remembered that we are multi-cellular organisms and that cell–cell communication is critical for health. Harnessing such knowledge and its subsequent application in repairing sporting injuries, may provide a more viable, effective, affordable long-term intervention programme, than stem cell transplantation alone or in combination with engineered scaffolds.

## Open Access

Open access for this article was enabled by the participation of Liverpool John Moores University in an all-inclusive *Read & Publish* pilot with Portland Press and the Biochemical Society under a transformative agreement with JISC.

## Abbreviations

ACI: autologous chondrocyte implantation
ESCs: embryonic stem cells
MACI: matrix-induced ACI
PRP: platelet rich plasma
RCTs: randomised controlled trials
WADA: world anti-doping agency
