# Supplementary material for: Stem cells and regenerative medicine in sport science
Source: Emerg Top Life Sci. 2021 Aug 27;5(4):563–73. doi: 10.1042/ETLS20210014 (PMC8589434; doi:10.1042/ETLS20210014)
Supplement: Supplementary Figures S1-S3 [file ETLS-5-563-s1.pdf]

Figure Legends:

Figure 1: Illustration of the potential and the potency of embryonic and adult stem cells. Created with BioRender.com

Figure 2: Illustration of the influence of exercise on adult stem cell expansion. Created with BioRender.com

Figure 3: Illustration of the development of interventions for use in sporting injuries (e.g. cartilage damage) from stem cells to bioengineering. Created with BioRender.com
